# Bisegmentectomy and venous reconstruction after portal vein embolization for the remnant hemiliver in a patient with recurrent colorectal liver metastases

**DOI:** 10.1002/ags3.12393

**Published:** 2020-08-29

**Authors:** Ryota Matsuki, Hirokazu Momose, Masaharu Kogure, Yutaka Suzuki, Yoshihiro Sakamoto

**Affiliations:** ^1^ Department of Hepato‐Biliary‐Pancreatic Surgery Kyorin University Hospital Mitaka Japan

**Keywords:** colorectal liver metastasis, future liver remnant, portal vein embolization, segmentectomy, venous reconstruction

## Abstract

Repeat hepatectomy for recurrent colorectal liver metastases (CRLM) for the remnant hemiliver is sometimes challenging due to the insufficient future liver remnant (FLR) volume. We present an aggressive strategy for resection of the recurrent CRLM involving bisegmentectomy of the remnant right hemiliver with the aid of portal vein embolization (PVE) and venous reconstruction. The patient was a 50‐year‐old woman who had undergone left hemihepatectomy for a CRLM 10 months ago. Three metastatic tumors were found in the remnant segments 7 and 8 (S7&8) of the liver, and one of them involved the right hepatic vein (RHV). Conducting bisegmentectomy of S7&8 with resection of the RHV, the non‐congestive FLR volume was calculated as 34.9% of the remnant total liver volume, which was deemed insufficient considering the mild liver damage after repeated chemotherapy. After trans‐ileocecal PVE of the portal branches in S7&8 in a hybrid angio room, the non‐congestive FLR volume increased to 42.3%, which could be further advanced to 58.0% if the RHV was reconstructed. Segmentectomies of S7&8 with resection and reconstruction of the RHV using the right superficial femoral vein graft was performed. The patient was discharged without any complications, and the postoperative computed tomography (CT) scan showed the good patency of the reconstructed venous graft. Aggressive segmentectomies and venous reconstruction of the remnant hemiliver after PVE might be a new strategy to overcome the insufficient FLR volume.

## Graphical abstract

Bisegmentectomy for recurrent colorectal liver metastases (CRLM) is sometimes challenging due to the insufficient future liver remnant volume in patients undergoing hemihepatectomy for initial CRLM. Segmentectomy combined with venous reconstruction after portal vein embolization for the remnant hemiliver could be a new strategy.

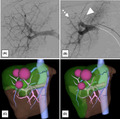

## 1 INTRODUCTION

Surgical resection remains the mainstay for cure the colorectal liver metastases (CRLM) even in the era of new anticancer chemotherapy. The recurrence rate of CRLM after initial hepatectomy has been reported to be 50%‐70%,^1^ and re‐resection would be crucial to obtain long‐term survival, because the time to surgical failure determines the life expectancy of each patient.^2^ Although parenchymal‐sparing hepatectomy rather than major hepatectomy would be preferable to treat patients with CRLM in order preserve the future liver remnant (FLR) volume,^3^ hemihepatectomy would often be required for the initial surgical resection, and major hepatectomy or segmentectomy for the recurrent CRLM is sometimes challenging due to the insufficient FLR volume. In addition, induction of perioperative oxaliplatin‐based chemotherapy increases the risks of post‐hepatectomy liver failure (PHLF).^4^ Here, we present an aggressive strategy for resection of the recurrent CRLM involving bisegmentectomies of the remnant hemiliver after trans‐ileocecal portal vein embolization (PVE) with reconstruction of the major hepatic vein.

## 2 PATIENT AND SURGICAL TECHNIQUE

**FIGURE 1:**
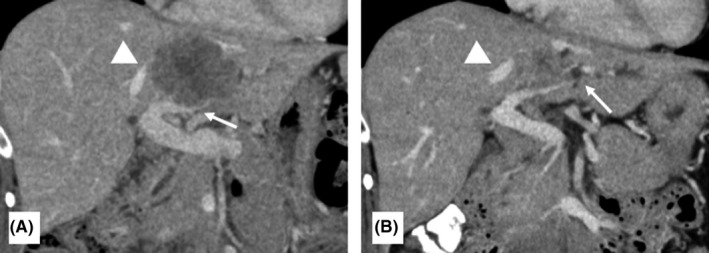
A, B, Contrast‐enhanced CT before first hepatectomy for colorectal liver metastases. A, Before neoadjuvant chemotherapy. Metastatic tumor in segment 2 &4 was involved the left glissonean pedicel (white arrow) and the main trunk of the middle hepatic vein (arrow head). B, After neoadjuvant chemotherapy. Metastatic tumor decreased the size, however, involvement of the left glissonean pedicle remained and the intrahepatic bile ducts in the segment 2 was dilated (white arrow). The tumor was still close to the main trunk of the middle hepatic vein (arrow head)

**FIGURE 2:**
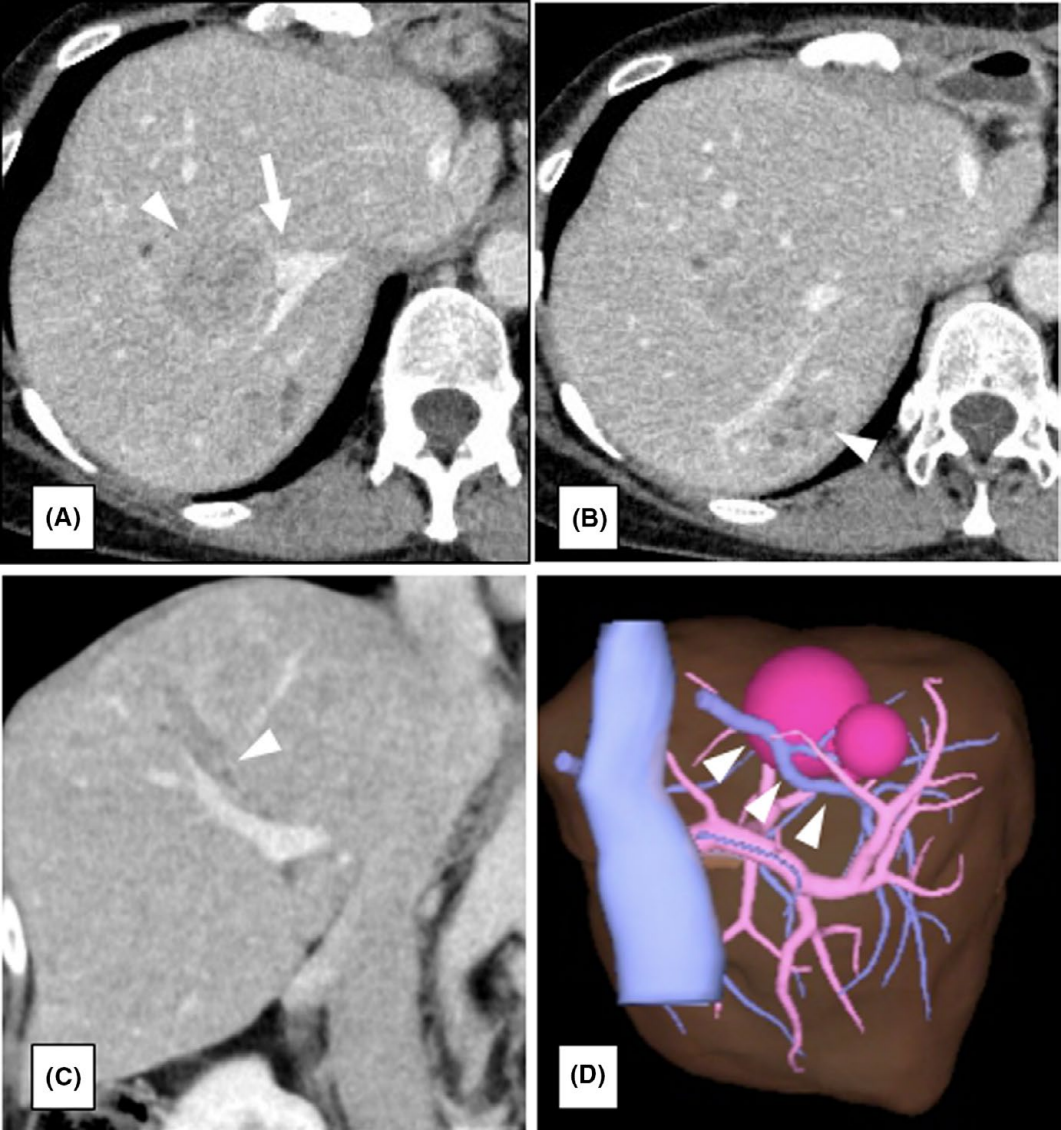
A, B, Contrast‐enhanced CT before portal vein embolization. Metastatic tumors located at segment 7 & 8 in the remnant liver (arrowhead). One of the metastatic tumor in segment 8 involved the right hepatic vein (arrow). C, Glissonean invasion of the main tumor in segment 8. The arrow head shows the glissonean invasion of the main tumor in segment 8. D, The 3D image of the liver. The main tumor in segment 8 involved the right hepatic vein for 4 cm in length (arrow head)

**FIGURE 3:**
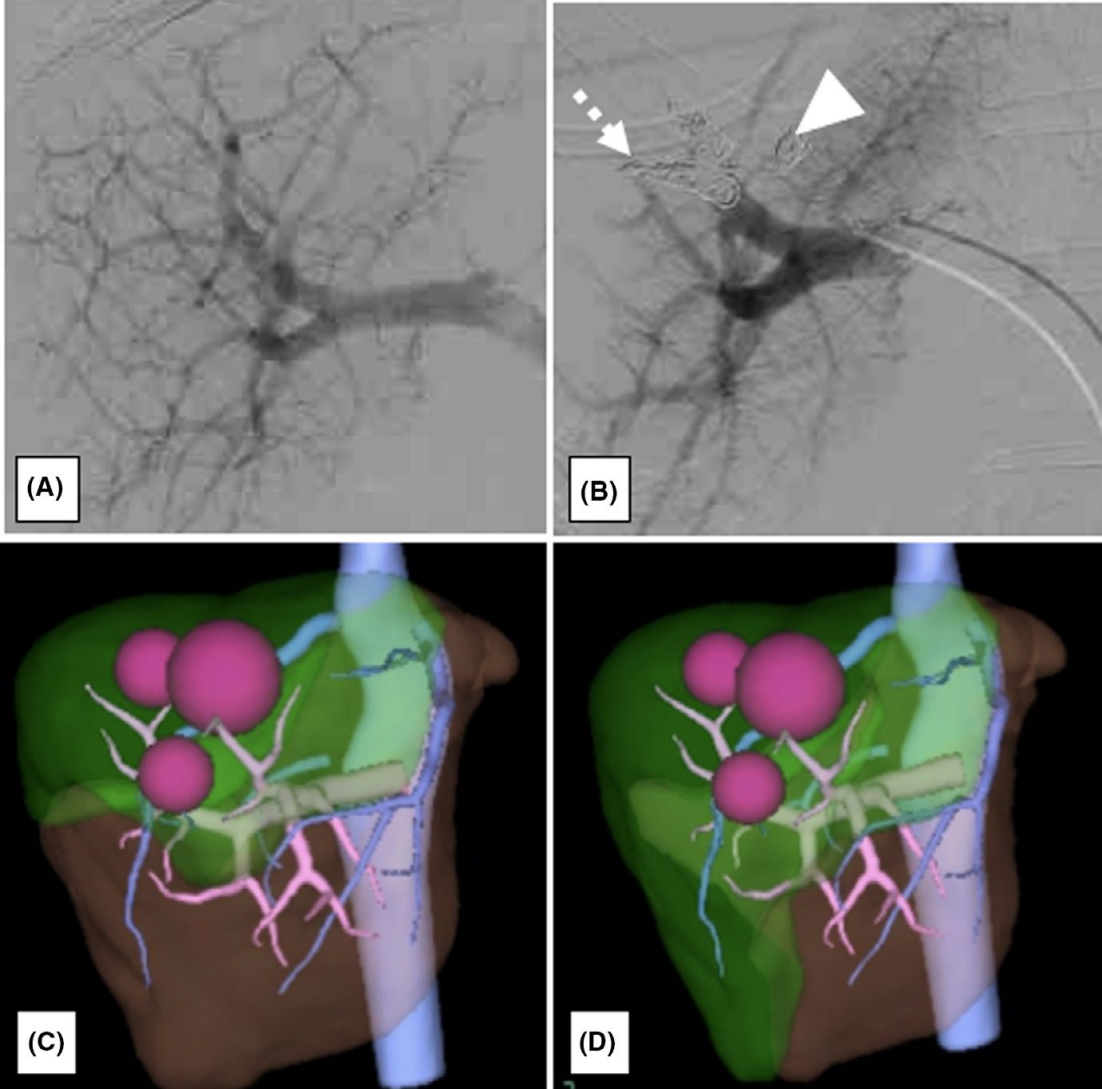
A, Portography before portal vein embolization (PVE). B, Portography after PVE. Portal venous branches of segment 8 (arrow) and 7 (arrowhead) were embolized using gelform and coils. C, The 3D‐simulation of the resection of the segment 7 & 8 with right hepatic vein (RHV) resection & reconstruction. Non‐congestive future liver remnant (FLR) was 58.0% of the total liver volume. D, The 3D‐simulation of the resecti on of the segment 7 & 8 with RHV resection & no reconstruction. Non‐congestive FLR was 42.3% of the total liver volume

**FIGURE 4:**
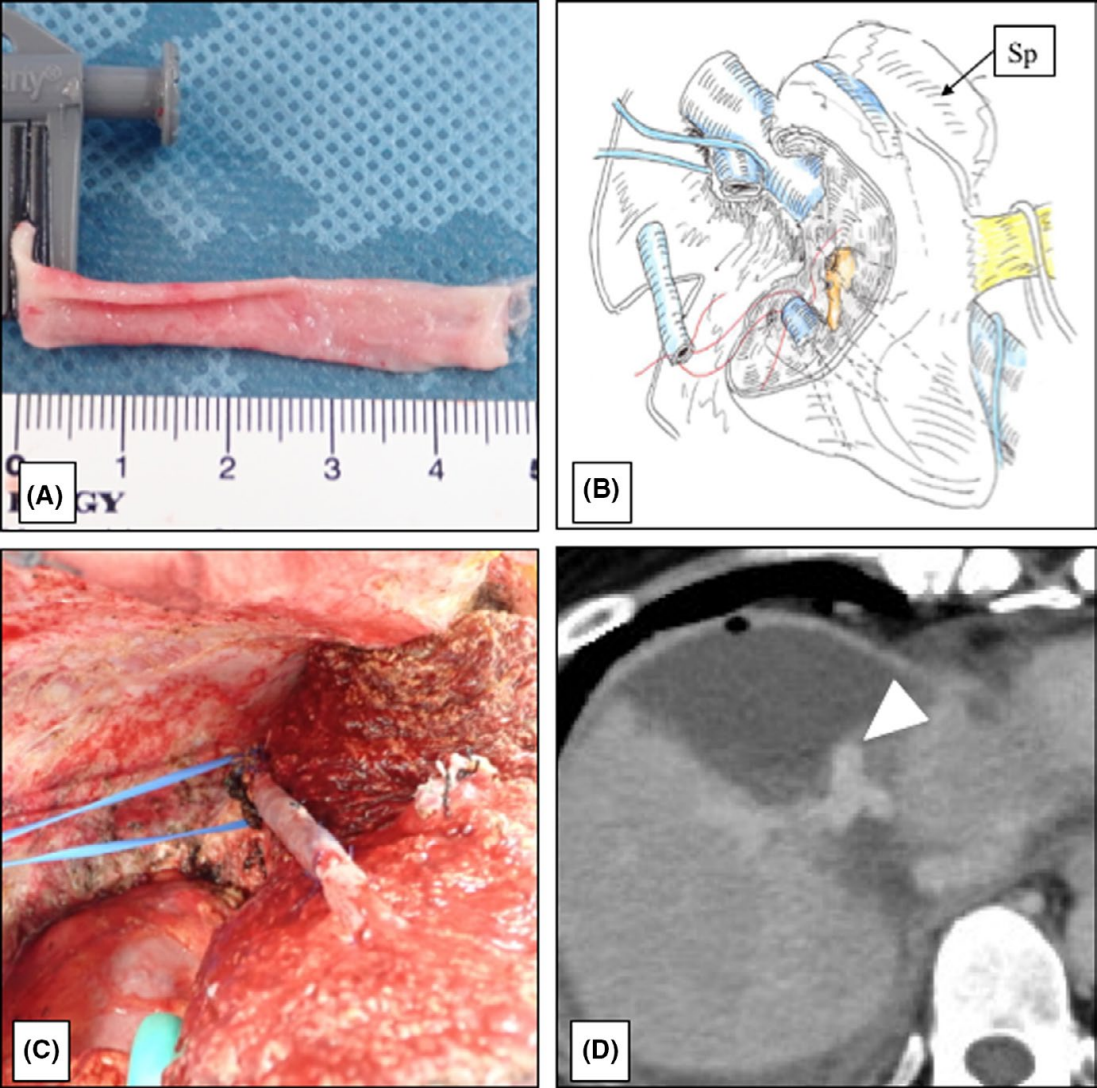
A, Harvested superficial femoral vein graft, sized 8 mm in diameter and 6 cm in length. B, Schema of the reconstruction of the right hepatic vein using the venous graft. Sp: Spiegel lobe of the remnant liver. C, After reconstruction of the right hepatic vein using the superficial femoral vein graft. D, Contra‐enhanced CT on day 4 after surgery. Contrast‐enhanced CT showed that although the reconstructed venous graft showed bending, patency was good (arrowhead)

The patient was a 50‐year‐old woman who underwent low anterior resection for rectal cancer and left hemihepatectomy for a synchronous liver metastasis in segments 2 and 4 involving the root of the glissonean pedicle of segment 2 after neoadjuvant chemotherapy using mFOLFOX6 plus panitumumab (Figure 1A,B). Contrast‐enhanced computed tomography (CT) showed three metastatic tumors in the remnant segments 7 and 8 of the liver 3 months after initial hepatectomy. As one of the tumors in segment 8 involved the right hepatic vein (RHV) and surgical resection could not be recommended (Figure 2A,B), systemic chemotherapy using mFOLFOX6 plus panitumumab was re‐administrated for 5 months. The maximum size of the tumors showed 7% reduction after chemotherapy, suggesting stable disease based on the RECIST 1.1 criteria.^5^ The emergence of drug allergy for oxaliplatin made it difficult to continue the chemotherapy by mFOLFOX6. The main tumor in segment 8, sized 5.2 cm in diameter, showed glissonean invasion along the glissonean pedicle of segment 8 on the dynamic CT scan (Figure 2C). Another tumor in segment 7, sized 3.7 cm in diameter, was free from the root of the glissonean pedicle of segment 7. However, the RHV having a thick branch from segment 7 was involved by the tumor in segment 8 for 4cm in length (Figure 2D). Thus, en bloc anatomical resection of S7&8 with resection of the RHV and its branch from segment 7 was deemed necessary to obtain adequate surgical field to reconstruct the RHV. Non‐congestive FLR volumes after bisegmentectomy of S7&8 combined with and without RHV reconstruction were calculated as 49.0% and 34.9%, respectively. As indocyanine green retention rate after 15 minutes (ICG‐R15) was 13.1%, suggesting moderate liver damage after repeated chemotherapy, 49.0% of FLR volume were insufficient according to the safe criteria of extensive liver resection, which recommends to preserve 50% of non‐congestive FLR when ICG‐R15 value is between 10% and 15%.^6^ Thus, we planned the resection of S7&8 with resection of the RHV after preoperative PVE to avoid PHLF. Preoperative PVE was performed via trans‐ileocecal vein approach (trans‐ileocecal portal embolization, TIPE) by interventional radiologists using three‐dimensional (3D) subtraction imaging in the hybrid angio room (Figure 3A,B). On 3D simulation of the liver resection two weeks after PVE, the non‐congestive FLR volume increased to 42.3% of TLV, which could be advanced up to 58.0% if the RHV was reconstructed (Figure 3C,D). The operation was performed 4 weeks after the TIPE. We performed resection of S7&8 combined with resection and reconstruction of the RHV using the right superficial femoral vein (SFV) graft. The SFV graft, sized 8 mm in diameter and 6 cm in length, was harvested before initiation of the liver transection (Figure 4A). Liver parenchymal transection was done using the clamp crushing method with vessel‐sealing device under intermittent Pringle's maneuver. After division of the liver parenchyma surrounding the RHV, the RHV was divided at the end of liver transection, and the hepatic specimen was extracted. After clamping of the RHV, no communication between the tributaries of RHV and middle hepatic vein (MHV) was found. The venous graft was used to interpose the defect of the RHV (Figure 4B,C). The operative time was 8 hours, and the estimated blood loss was 800 mL. Postoperative course was uneventful, and the edema of the right lower leg associated with harvesting the superficial femoral vein was mild and reversible (18% increase of the circumference on day 8 in maximum). Contrast‐enhanced CT on day 4 showed a good patency of the graft (Figure 4D). The patient was discharged on day 15 without any sign of PHLF according to the International Study Group of Liver Surgery^7^ or other complications. Pathological findings revealed that the metastatic tumor invaded the wall of the RHV, but the surgical margin was negative for cancer.

## 3 DISCUSSION

Although repeat hepatectomy for recurrent CRLM is crucial for the survival of patients,^2^ it is sometimes challenging when the remnant FLR volume is small. Multiple CRLM recurrence in the hemiliver involving the major hepatic veins is often considered to be unresectable because of the insufficient FLR volume. To expand the surgical indication for recurrent patients with small FLR volumes, preoperative PVE has been widely used for the purpose of increasing the FLR volume in a few weeks. This ancillary procedure has been utilized before major initial hepatectomies for hilar cholangiocarcinoma or multiple CRLM.^8^ Reports on selective PVE for Couinaud's segments are rare. Kishi et al at the MD Anderson Cancer Center described the efficacy of selective embolization of P4 in addition to the embolization of the right portal vein.^9^ Care should be taken to not embolize the branches in segments 2 and 3. Hiramatsu et al at Nagoya University reported the selective PTPE of the left lateral sectional branches for CRLM after right hepatectomy.^10^ After PTPE, they successfully performed left lateral sectionectomy, preserving segment 4. However, in a vast review of the literature, we could not find the selective embolization of the portal branches in S7&8. Thus, this is the first report of the selective PVE for S7&8 for the remnant right hemiliver. Nowadays, PTPE rather than TIPE would be the first choice of PVE. However, PTPE of P7 and 8 of the remnant right hemiliver is technically demanding because reverse retrograde embolization under local anesthesia is required. TIPE in a hybrid angio room under general anesthesia makes it possible to perform selective antegrade embolization. In the present case, TIPE was successfully performed and considerable hypertrophy (7.4% of the TLV) of the FLR volume was obtained two weeks after the TIPE.

Resection of the major hepatic vein will evoke the congestion of the corresponding areas, and the portal uptake function of these areas was reported to be 10%‐80% depending on the frequency of inherent collaterals.^11^ The frequency of inherent collaterals between RHV and MHV was reported to be only 14% in humans.^12^, ^13^ However, it's actually difficult to know the development of the inherent venous collaterals based on the preoperative imaging studies. Thus, to avoid PHLF, it is necessary to estimate the functional FLR volume excluding the congestive areas. In the present case, the non‐congestive FLR volume without venous reconstruction was 42.3%, which was deemed insufficient considering the damaged hepatic function (ICG‐R15 = 15% was after TIPE) caused by repeated chemotherapy, and we planned to perform venous reconstruction because no communication between the tributaries of RHV and MHV was found.

Reconstruction of the major hepatic vein will expand the indication of extensive hepatectomy for CRLM,^14^, ^15^ and several kinds of venous grafts, such as the iliac vein, the internal jugular vein, the left renal vein, the ovarian vein, the great saphenous veins, and the homograft, had been used for replacing the venous defects.^16^ Sacrificing the iliac vein would be associated with the postoperative edema of the hemi‐leg, which continues 1‐3 months after surgery.^17^ Although the present superficial femoral vein graft is slightly thinner than the iliac venous graft, it is fully available for reconstruction of the major hepatic or portal veins, and the postoperative congestion of the lower leg is mild, because the deep femoral vein and the great saphenous vein are preserved. In this case, the edema of the leg that was harvested SFV was also mild and disappeared 2 months after surgery.

Associating liver partition and portal vein ligation for staged hepatectomy (ALPPS) might be an alternative to rapidly increasing the FLR volume and expanding the surgical curability. This is a new two‐stage hepatectomy procedure and might be a breakthrough to overcome the insufficient FLR volume. The hypertrophy of FLR volume in ALPPS is rapid, but the mortality rate has been reported to be 9% after resection for CRLM and 27% for perihilar cholangiocarcinoma.^18^ To increase the safety of the original ALPPS, we have introduced partial TIPE ALPPS not only for CRLM but for perihilar cancer,^19^ in which the partial division of the liver parenchyma and TIPE were performed during the first stage, instead of total division combined with resection of the middle hepatic vein. Lesser invasiveness of the first stage operation was associated with lesser surgical morbidity. However, the original ALPPS would be inappropriate for resection of S7&8 because it is impossible to ligate the individual intrahepatic third‐order portal branches. Thus, classical TIPE would be the better and safer method to increase the FLR volume for the remnant hemiliver.

In conclusion, we present a new strategy for resection of the CRLM involving bisegmentectomy with an aid of TIPE and venous reconstruction using an autologous venous graft customized from the SFV, in a patient with mild liver damage after repeated chemotherapy. We believe it will be useful to overcome the insufficient FLR and to expand the surgical indication for patients with recurrent CRLM.

## DISCLOSURE

Funding: None.

Conflict of Interest: The authors have no competing interests to declare.

Author Contribution: Study conception and design: Yoshihiro Sakamoto; Acquisition of data: Ryota Matsuki, Yutaka Suzuki, Masaharu Kogure, Hirokazu Momose; Analysis and interpretation of data and Drafting of manuscript: Ryota Matsuki; Critical revision of manuscript: Yoshihiro Sakamoto.

## ETHICAL STATEMENTS

All authors have seen and approved the final version of the manuscript being submitted, and all authors fulfill the COPE (Committee on Publication Ethics) requirements for authorship.
